# Editorial: Integrating molecular mechanisms, immunotherapy, and drug sensitivity in cancer immunology and oncology

**DOI:** 10.3389/fimmu.2026.1933749

**Published:** 2026-07-22

**Authors:** Weize Sun, Guangming Yu, Zhengyuan Fang, Yuan Qian, Zhiwen Luo, Qizhi Liu

**Affiliations:** 1 Huashan Clinical Medical College, Fudan University, Shanghai, China; 2 Department of Orthopedics, Dexing Traditional Chinese Medicine Hospital, Dexing, Jiangxi, China; 3 Department of Gastrointestinal Surgery, Renji Hospital, School of Medicine, Shanghai Jiao Tong University, Shanghai, China; 4 Nanjing Hospital of Chinese Medicine Affiliated to Nanjing University of Chinese Medicine, Nanjing, Jiangsu, China; 5 Department of Sports Medicine, Huashan Hospital, Fudan University, Shanghai, China

**Keywords:** cancer immunology, drug sensitivity, editorial, immunotherapy, integration, molecular mechanisms, oncology

**Figure 1:**
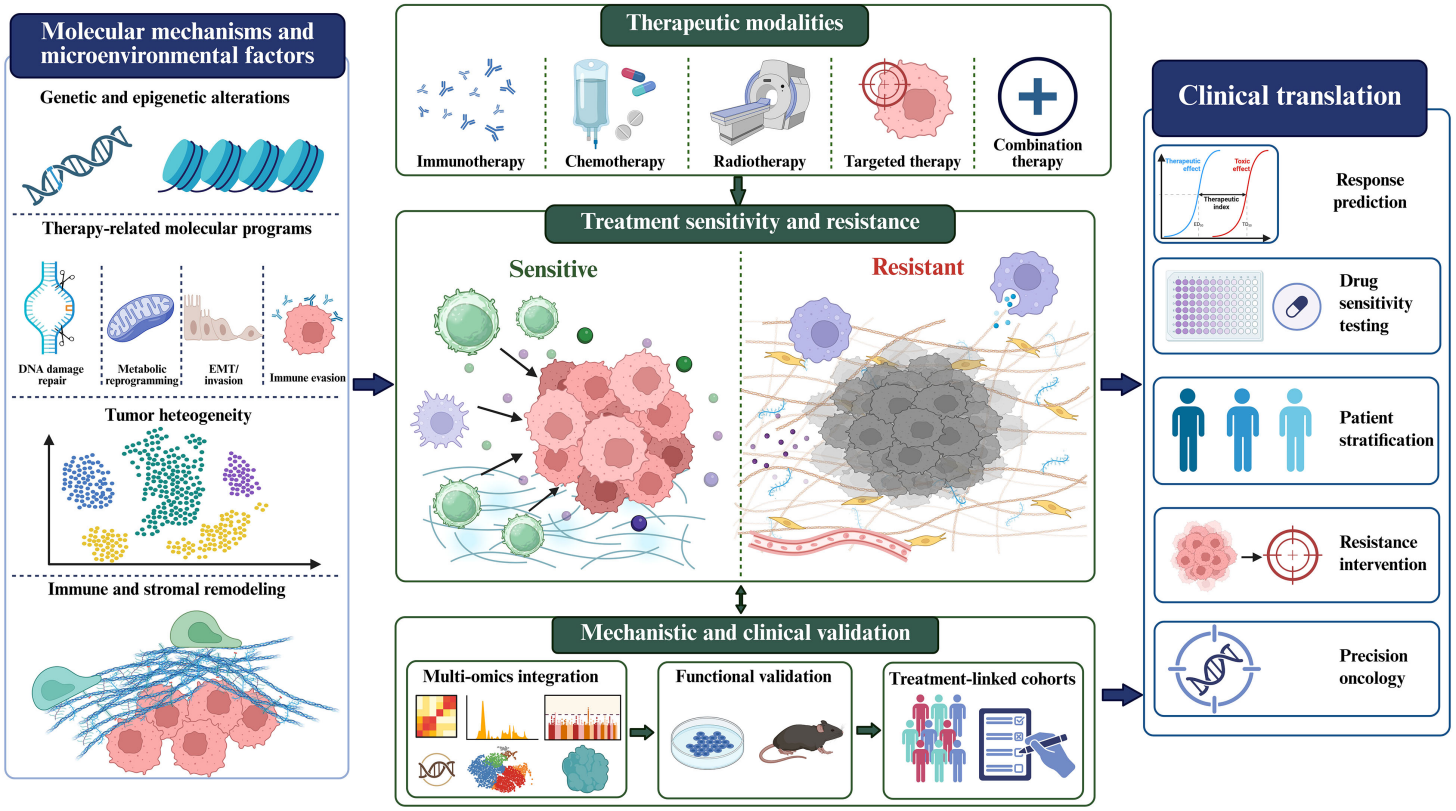
From molecular context to therapeutic response in cancer immuno-oncology.

With the development of precision oncology, the research focus has gradually shifted from acquiring tumor molecular profiles to interpreting molecular information in clinically meaningful ways. Tumor progression and treatment failure are not determined by a single genetic alteration alone, but are closely associated with tumor heterogeneity, the immune microenvironment, stromal architecture, and therapy-induced changes in cellular states (1–4). Immune checkpoint therapy and large-scale drug sensitivity studies further indicate that molecular classification can inform therapeutic decision-making only when it is connected to treatment response, functional validation, and clinical cohorts (5, 6). Recent studies also show that extracellular matrix remodeling can influence immune-cell infiltration and is associated with responses to immune checkpoint blockade (7, 8). Extending this line of evidence, the associations of intratumoral collagen deposition with angiogenic states and of exercise-responsive microRNAs with responses to anti-B7-H3 therapy further suggest that microenvironmental architecture and host-related factors may jointly regulate sensitivity to immunotherapy (9, 10). The articles collected in this Research Topic cover diverse tumor types and treatment settings, and together show that molecular findings require mechanistic investigation, functional validation, and clinical evidence before they can inform decisions about immunotherapy, chemotherapy, radiotherapy, or targeted therapy. As summarized in **Figure 1**, molecular mechanisms and microenvironmental factors jointly shape treatment sensitivity and resistance, whereas multi-omics integration, functional validation, and treatment-linked cohorts support response prediction, patient stratification, drug sensitivity assessment, resistance intervention, and precision oncology.

Several clinical studies directly address treatment selection and ask whether molecular biomarkers or combination strategies can influence therapeutic decisions. In recurrent or metastatic head and neck squamous cell carcinoma, Jiang et al. prospectively evaluated 11q13 amplification as a decision-making biomarker for first-line anti-PD-1 therapy. Treatment was assigned according to 11q13 status, and an external control group of patients with 11q13 amplification receiving anti-PD-1 therapy was included, allowing the study to directly test whether this biomarker could be incorporated into treatment stratification. The favorable response and survival outcomes observed in patients without amplification support this direction, although the single-center, non-randomized design indicates that larger cohorts are still needed for validation. Chen et al. provided multicenter real-world evidence for cadonilimab in recurrent or metastatic cervical cancer, including efficacy, safety, PD-L1 testing, and whole-exome sequencing. The study reflects patients treated in routine clinical settings rather than highly selected trial populations. Because it lacks a randomized control group, its findings are best viewed as complementary evidence on drug activity and safety rather than as a basis for defining the optimal treatment sequence. Zhong et al. used propensity score matching to evaluate preoperative TACE combined with PD-1 inhibitors and tyrosine kinase inhibitors in hepatocellular carcinoma beyond the up-to-seven criteria. After matching, recurrence-free survival was prolonged without an increase in severe complications, providing a rationale for prospective evaluation of perioperative treatment strategies in this high-risk population. Li et al. found no significant difference between HER2-low and HER2–0 early breast cancers in sensitivity to neoadjuvant chemotherapy or overall prognosis. This result indicates that HER2-low status, although therapeutically relevant in the era of antibody-drug conjugates, should not be directly extrapolated as a predictor of conventional chemotherapy sensitivity.

These clinical studies also underline the need to distinguish prognostic associations from predictive evidence. A biomarker that separates survival curves is not necessarily ready to guide treatment selection. Likewise, activity observed for a combination therapy in real-world data needs to be separated, through rigorous study design, from the effects of patient selection, treatment sequence, and center-level differences. For a Research Topic focused on drug sensitivity, this distinction is important because clinical application ultimately depends on treatment-specific predictive value rather than general risk stratification.

In mechanistic studies, the central question is whether a molecular finding is associated with a measurable change in drug response. Jia et al. linked IRX4 hypermethylation and reduced expression to colorectal cancer progression and oxaliplatin sensitivity, and supported this relationship through pyrosequencing, expression analyses, cell-based functional assays, drug sensitivity testing, and analysis of the NF-kB/EGFR signaling axis. Gao et al. identified responsive and resistant tumor states in colorectal cancer treated with neoadjuvant chemotherapy, proposed GDC-0941 through high-throughput drug screening, and used animal models to support the role of the KLF5/PI3K/AKT axis in restoring oxaliplatin sensitivity. Both studies connect resistant states with experimentally induced changes in drug response, moving closer to translational research requirements than studies that only describe cell clusters, methylation events, or risk scores.

Studies of DNA damage and radiotherapy can be considered through a similar logic. Dong et al. established a carboplatin-resistant three-dimensional breast cancer model, developed a DNA damage repair-related gene signature, and connected it with prognosis, immune infiltration, and sensitivity to DNA-damaging agents. Functional experiments involving TONSL provided support beyond database-based analysis. Yu et al. performed a high-content clonogenic survival screen of FDA-approved drugs and identified maytansine as a potent radiosensitizer for meningiomas. Clonogenic survival reflects the sustained proliferative capacity of tumor cells after irradiation and is therefore a suitable functional endpoint for radiosensitizer screening. Subsequent analyses of DNA double-strand breaks, G2/M arrest, transcriptomic changes, and *in vivo* experiments further strengthened the findings. Huang et al. connected PRKDC, cGAMP-mediated innate immune activation, macrophage polarization, and nanodelivery in lung cancer models, showing that innate immune signaling may provide an intervention point for enhancing drug sensitivity. Ayinde et al. focused on the therapeutic product itself by characterizing a bispecific antibody targeting EGFR and VEGF-A. For such complex biologics, target selection is only the starting point; binding activity, signaling blockade, and paracrine effects mediated by each arm require separate validation.

Drug sensitivity is influenced by intrinsic states of malignant cells as well as by immune and stromal cells. Jiang et al. constructed a pan-cancer single-cell atlas of IFI30-positive macrophages and supported the computational findings with coculture experiments, bringing antigen processing and macrophage states into the discussion of immunotherapy response. Liang et al. integrated single-cell RNA sequencing, spatial transcriptomics, bulk transcriptomics, machine learning, and coculture experiments in bladder cancer, and proposed that dysregulated taurine metabolism promotes immunosuppression and anti-PD-1 resistance. Qin et al. examined taurine metabolism-related mechanisms in pancreatic cancer, identifying RPS4Y1-positive tumor cells, fibroblast-CD8 T cell-tumor cell crosstalk, spatial colocalization, and LY6D as a candidate target. These two taurine-related studies analyze metabolic abnormalities within the interactions among malignant cells, fibroblasts, macrophages, and cytotoxic lymphocytes. They have not yet shown that targeting these programs improves patient outcomes, but they define more specific cell types and molecular candidates for future intervention studies.

Single-cell studies further refine the analysis of tumor heterogeneity and intercellular interactions, although candidate targets derived from cellular atlases still require functional validation. Zhao et al. identified a C2 IGF2-positive tumor cell subpopulation in high-grade serous ovarian cancer and linked it to malignant progression, fibroblast communication, metabolism, immune infiltration, and predicted drug sensitivity. Song et al. identified C5 PCOLCE-positive tumor cells in uveal melanoma and validated that CITED1 promotes cell proliferation, invasion, and migration. Lin et al. used colorectal cancer single-cell data to identify FXYD5-positive tumor cells and validated DLX2 *in vitro*, suggesting that this subpopulation and its associated genes may contribute to tumor progression and immunotherapy stratification. In osteosarcoma, Zhang et al. combined EMT-related modeling, immune response prediction, single-cell analysis, and GPC3 knockdown experiments. The review by Shi et al. on NF-kB signaling and the osteosarcoma microenvironment adds mechanistic context regarding macrophage and myeloid-derived suppressor cell recruitment, PD-L1 regulation, anti-apoptotic signaling, and therapeutic resistance. These studies show that single-cell atlases can resolve tumor heterogeneity into more specific cellular states and communication programs, but their therapeutic relevance still depends on whether perturbation experiments and treatment-linked cohorts can show that these states alter drug or immunotherapy response.

Biomarker and multi-omics studies represent a substantial part of this Research Topic. The components with greater translational relevance are those that move beyond risk stratification and connect a score or candidate gene with immune mechanisms, treatment response, or functional experiments. Lu et al. studied CXCR6 in muscle-invasive bladder cancer using TCGA, GEO, a single-center cohort, the IMvigor210 immunotherapy cohort, and single-cell data, linking high CXCR6 expression with the CXCR6-CXCL16 axis-mediated interaction between T/NK cells and myeloid cells and with improved anti-PD-L1 treatment response. This provides both biological and clinical support for CXCR6 as a biomarker. Zeng et al. studied SLFN11 in melanoma and incorporated assays of macrophage polarization, immune cell recruitment, and CD8 T cell cytotoxicity, extending SLFN11 from a prognostic marker to a factor linked with immune microenvironment function. Whether it can guide treatment selection still requires pretreatment and post-treatment cohorts as well as prospective validation. Xie et al. integrated bulk RNA sequencing, single-cell analysis, spatial transcriptomics, metabolic inference, machine learning, and qRT-PCR validation to link ribosome biogenesis with clear cell renal cell carcinoma progression, immune dysfunction, and tolerance to targeted therapy. The study suggests that some tumors may show increased immune-related signals while effective antitumor immunity remains suppressed, a state closer to the real complexity of the microenvironment than a simple division into immunologically hot or cold tumors. However, further work is needed to determine whether this score can affect treatment selection. Pei et al. proposed a coagulation-related classification in colorectal cancer and pan-cancer and used TIMP1 experiments to suggest that coagulation biology may influence immune states and treatment response. Wang et al. developed a liquid-liquid phase separation-related gene signature in colon cancer and validated POU4F1 *in vitro*. The shared value of these studies lies in linking high-throughput models with immune context, candidate mechanisms, or functional experiments. They also indicate that the clinical value of multi-omics and machine learning models should not remain at the level of dividing patients into high- and low-risk groups. When a score is stable across platforms, predicts response in a population receiving a specific therapy, and points to mechanisms that can be tested in cells, organoids, animal models, or paired clinical samples, patient stratification becomes more likely to enter treatment-oriented research. Several studies in this Research Topic move in this direction, although relatively few complete the full path from model construction to therapeutic validation.

Candidate gene studies in this Research Topic often begin with public datasets and then add clinical samples or functional experiments. Chen et al. identified a malignant epithelial cell subpopulation in gastric cancer and supported ANXA5 as an oncogenic factor associated with invasion, EMT, prognosis, and drug tolerance. Xu et al. developed an m7G methylation-related prognostic signature in head and neck squamous cell carcinoma and validated LARP1 using clinical samples and knockdown experiments. Zhao et al. combined single-cell analysis, cell experiments, and animal experiments to position NUSAP1 as a diagnostic, prognostic, and mechanistically relevant marker in glioma. Zhong et al. explored SUSD3 across cancers, combining immune-related analyses, drug sensitivity prediction, molecular docking, and breast cancer cell experiments. Pan-cancer analysis is useful for hypothesis generation, but the parts that add greater clinical credibility remain those focused on specific tumor types and supported by functional perturbation. Wang et al. used cross-disease transcriptomic analysis to propose that DOK3 and PAPOLA may connect neuroinflammation and tumorigenesis, and validated DOK3 in glioblastoma and microglial cell models. This study suggests that inflammatory and oxidative stress programs may affect neural injury, immune regulation, and tumor biology, while also showing that cross-disease analyses require experimental systems to define the specific functions of candidate genes.

Review articles provide the necessary mechanistic background for these original studies. Xu et al. reviewed intrinsic and microenvironmental drivers of drug resistance in gastrointestinal cancers, including drug-target alterations, drug efflux, DNA repair, epigenetic remodeling, EMT, stromal effects, organoids, liquid biopsy, and AI-assisted analysis. Zhao et al. focused on mitochondrial dysfunction in colorectal cancer progression and drug resistance, aligning with the metabolic and drug sensitivity-related studies in this Research Topic. Kang et al. reviewed epigenetic regulation of immune escape in breast cancer. Tang et al. discussed the role of OTU family deubiquitinases in tumor signaling and immune escape. Cao et al. considered Beclin-1 at the intersection of autophagy, apoptosis, ferroptosis, treatment response, and tumor immunity. These reviews show that treatment sensitivity and resistance are regulated by multiple pathways and vary by tumor type, treatment timing, and immune context.

Other reviews place these questions in settings closer to clinical practice. Huang et al. summarized drug tolerance and immune escape in bladder cancer, with particular attention to genetic and epigenetic alterations, the tumor microenvironment, PI3K/AKT and MAPK signaling, and combination therapy. Huang et al. discussed NK cell exhaustion in hepatocellular carcinoma and proposed combining checkpoint blockade with strategies that restore NK cell cytotoxicity. Sun et al. summarized therapeutic strategies for adrenocortical carcinoma, covering Wnt/beta-catenin, IGF2/IGF1R, p53-related apoptosis, mitotane, targeted therapy, and immune checkpoint inhibitors, and emphasized the need for molecularly stratified clinical studies in rare tumors. Wang et al. discussed sepsis-driven metabolic reprogramming and its effects on immunotherapy, metastasis, and drug sensitivity, bringing infection and systemic inflammatory status into the framework of precision treatment.

Taken together, this Research Topic places molecular mechanisms, immunotherapy, and drug sensitivity within a shared translational problem: how to convert detectable molecular or cellular states into verifiable treatment prediction. The collected studies show that biomarkers need to demonstrate predictive value in patients receiving the relevant therapy, differentially expressed pathways require perturbation experiments showing that they can alter drug response, and combination therapies should be designed around measurable resistance mechanisms. Future research will require more independent cohorts, paired pretreatment and post-treatment samples, organoid models, and animal models to connect molecular findings, functional results, and clinical responses more closely. On this basis, molecular oncology, immunotherapy, and drug sensitivity analysis can be translated more reliably into patient stratification, treatment selection, and strategies to overcome therapeutic resistance.
